# Stromal fibroblasts derived from mammary gland of bovine with mastitis display inflammation-specific changes

**DOI:** 10.1038/srep27462

**Published:** 2016-06-07

**Authors:** Qing Chen, Guiliang He, Wenyao Zhang, Tong Xu, Hongliang Qi, Jing Li, Yong Zhang, Ming-Qing Gao

**Affiliations:** 1 College of Veterinary Medicine, Northwest A&F University, Yangling 712100, Shaanxi, China; 2 Key Laboratory of Animal Biotechnology, Ministry of Agriculture, Northwest A&F University, Yangling 712100, Shaanxi, China

## Abstract

Fibroblasts are predominant components of mammary stromal cells and play crucial roles in the development and involution of bovine mammary gland; however, whether these cells contribute to mastitis has not been demonstrated. Thus, we have undertaken biological and molecular characterization of inflammation-associated fibroblasts (INFs) extracted from bovine mammary glands with clinical mastitis and normal fibroblasts (NFs) from slaughtered dairy cows because of fractured legs during lactation. The functional contributions of INFs to normal epithelial cells were also investigated by using an *in vitro* co-culture model. We present evidence that the INFs were activated fibroblasts and showed inflammation-related features. Moreover, INFs significantly inhibited the proliferation and β-casein secretion of epithelial cells, as well as upregulated the expression of tumor necrosis factor-α and interleukin-8 in epithelial cells. These findings indicate that functional alterations can occur in stromal fibroblasts within the bovine mammary gland during mastitis, demonstrating the importance of stromal fibroblasts in bovine mastitis and its treatment.

Mastitis is an inflammatory reaction of the mammary gland usually caused by microbial infection and is recognized as the most costly infectious disease of dairy cattle worldwide^1^. Tissue damage during mastitis can initially be caused by bacteria, followed by an influx of immune cells, such as polymorphonuclear neutrophils and macrophages, into the mammary gland^1^. Contact between bacteria invading the mammary gland with both somatic cells in the milk and with lining epithelial cells results in an innate immune response^2^. Resident and recruited immune cells, as well as mammary epithelial and endothelial cells, play important roles in the immediate defense against local infections by the production of cytokines and other inflammatory mediators^3^.

The mammary gland is a complex organ of various tissue and cell types, and epithelia cells and fibroblasts are predominant components of mammary parenchyma and stroma, respectively^4^,^5^. Fibroblasts play crucial roles in the development and involution of murine, human, and bovine mammary glands, as well as in lactation^6^,^7^,^8^. Accumulating evidence suggests that the fibroblast functions as a structural element and as a vital immunoregulatory cell^9^. In addition to their role as structural elements, fibroblasts could serve as resident sentinel cells involved in would healing and cleaning invading microorganisms; this function is activated by substances released during tissue injury, derived from infectious microorganisms, or caused by other environmental factors. Studies conducted over the recent decade have demonstrated that tissue-resident fibroblasts influence the persistence of the inflammatory lesion of organs^10^. With the stimulation of cytokines, which are primarily produced by monocytes/macrophages early in the inflammatory reaction, fibroblasts can synthesize and express an upregulated effector phenotype for some cytokines; these fibroblast-derived cytokines may play a significant role in the amplification of the inflammatory response^11^. The roles of fibroblasts in the regulation of immune responses have been clarified in the inflammatory reactions of colitis^12^, airway inflammation^13^, and tumor-enhancing inflammation of the mammary gland^14^.

During mastitis, macrophages and epithelial cells release chemoattractants, which trigger the migration of leukocytes from the blood towards the mammary gland^1^. Neutrophils^15^, macrophages^16^,^17^, lymphocytes^18^, and host proteases and cytokines contribute to mammary tissue damage during mastitis^19^; however, whether fibroblasts, the predominant cells of mammary stroma, contribute to bovine mastitis remains unknown.

This study aims to improve our understanding of the role of the mammary stromal fibroblasts in bovine mastitis by comparison of the biological characteristics of mammary stromal fibroblasts derived from dairy cows with and without mastitis. The comparison is based on cell morphology, proliferation, apoptosis, migration, global gene expression profiles, as well as the effects on the functions of mammary epithelial cells in an *in vitro* co-culture model. We demonstrate that mammary stromal fibroblasts derived from mastitic cows exhibit inflammation-specific changes compared with those from dairy cows without mastitis and represent their inflammatory potential. The findings of this study indicate the importance of examining the roles of fibroblasts within the mammary gland for mastitis therapy and studying the mechanisms of bovine mastitis.

## Results

### Isolation of fibroblasts and epithelial cells from bovine mammary tissues

**Figure 1:**
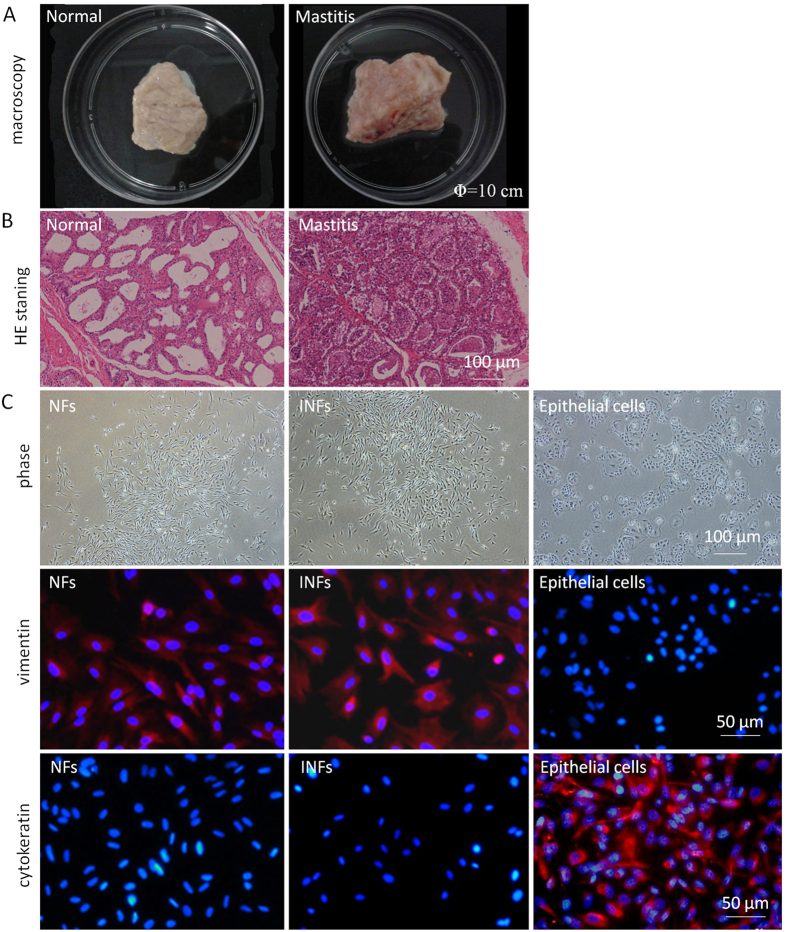
Collection of representative mammary tissue samples and extraction of fibroblasts and epithelial cells. **(A)** Normal and mastitic mammary tissues were distinguished by macroscopic examination, as described in the text. Φ: diameter of dishes. **(B)** The representative pathological appearance of normal and mastitic mammary tissues was confirmed by hematoxylin and eosin (H&E) staining. **(C)** Normal fibroblasts (NFs), inflammation-associated fibroblasts (INFs), and epithelia cells isolated from the tissues were examined by phase-contrast microscopy and immunostaining for vimentin or cytokeratin; nuclei were stained with DAPI. Both NFs and INFs exhibited an identical long and spindle-shaped morphology and vimentin expression and negative staining for cytokeratin. By contrast, epithelial cells showed cobblestone-like appearance, positive cytokeratin expression, and negative vimentin expression. All representative images were obtained from one of three independently repeated experiments.

Mammary gland tissues were acquired from Holstein dairy cows with or without mastitis by macroscopic dissection, followed by microscopic examination. For reproducible identification, only tissues that were confirmed by microscopic examination were included. Representative images of tissue sections of mammary gland from cows with or without mastitis can be seen in Fig. 1. The normal mammary tissue presented a regular cut surface and showed a uniform texture with few connective tissues, whereas the tissues from mastitic cows presented an irregular cut surface and a variable admixture of epithelium, abundant connective tissue, and blood clot in some cases (Fig. 1A). HE staining indicated that normal mammary parenchymal tissue exhibited large alveolar luminal areas with no evidence of inflammation, minimal cellular debris, and limited stromal area, whereas inflammation was characterized by massive neutrophil recruitment into the alveolar and tubular spaces in mammary tissue sections with mastitis (Fig. 1B).

The bacteriologic evaluation of the mastitic cows had revealed various pathogenic bacteria. In this study, only five mastitic cow samples in which *Escherichia coli* was the sole pathogen were used to extract fibroblasts. These fibroblasts were designated as inflammation-associated fibroblasts (INFs). Meanwhile, normal fibroblasts (NFs) were extracted from six cows without mastitis. Both INFs and NFs possessed the basic fibroblast characteristics of an identical and long spindle-shaped morphology under a phase-contrast microscope, strong expression of the fibroblastic marker vimentin, and negative staining for the epithelial marker cytokeratin (Fig. 1C). In addition, normal epithelial cells were isolated from the mammary glands of three cows and exhibited a cobblestone-like appearance, positive expression of cytokeratin, and negative expression of vimentin (Fig. 1C). These findings indicate that fibroblasts and epithelial cells were well separated from the other type of cells and are thus considered highly homogeneous with minimal contamination. NFs, INFs, or epithelial cells extracted from the aforementioned cows (six cows without mastitis for NFs, five mastitic cows for INFs, and three cows without mastitis for epithelial cells) were used as cell pools in all subsequent experiments. Each type of cell pool was prepared by mixing equivalent cells from all individuals in each group.

### Comparison of basic characteristics between INFs and NFs

**Figure 2:**
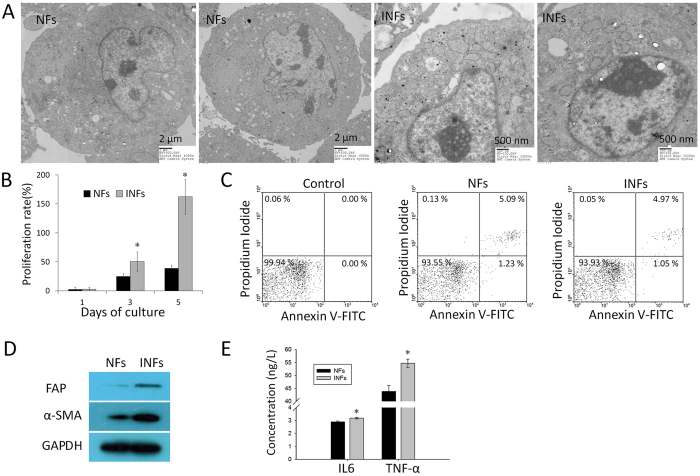
Comparison of the basic characteristics between NFs and INFs. **(A)** Images of NFs and INFs under a transmission electron microscope. **(B)** NFs and INFs seeded at the same density in 96-well plates were assayed for cell proliferation on indicated days after culture using Cell Counting Kit-8. Cell proliferation rates were calculated using the formula (OD_n _− OD_0_)/OD_0 _× 100 (OD, optical density value on indicated days after culture; n, days of culture). INFs exhibited significantly higher proliferation rates compared with NFs. *P < 0.01 versus NFs. **(C)** NFs and INFs grown to confluence were routinely collected, and apoptosis was measured by Annexin/PI staining and flow cytometry. The percentages of apoptotic cells were plotted, and a similar apoptotic rate was observed in NFs and INFs. **(D)** Expression of fibroblast activation protein (FAP) and alpha-smooth muscle actin (α-SMA) in NFs and INFs by Western blot analysis. INF expressed higher level of FAP and α-SMA than NFs. **(E)** NFs secreted increased interleukin 6 (IL6) and tumor necrosis factor- alpha (TNF-α) compared with NFs, as determined using ELISA kits. *P < 0.01 versus NFs. The pooled cells of NFs and INFs were measured in triplicate and all data were representative for one of three independently repeated experiments.

Numerous studies suggest that fibroblasts taken from diseased tissues have a fundamentally different phenotype compared with fibroblasts taken from normal tissues at the same anatomical site^20^. Transmission electron microscopy indicated that both INFs and NFs showed typical features of fibroblasts—extensive, well-developed rough endoplasmic reticuli, mitochondria, Golgi apparatus, and large nuclei with well-organized chromatins (Fig. 2A); however, INFs include more lysosomal vacuoles than NFs.

The fibrotic reaction of the connective tissue following an inflammatory response could result in the proliferation of local fibroblasts^21^. To investigate whether mastitis could enhance the proliferation of stromal fibroblasts, NFs and INFs were seeded in 96-well plates at the same density and then cultured for indicated days; the proliferation was then evaluated. The cell proliferation rate of INFs was higher than that of NFs on Days 3 and 5 post-culture (Fig. 2B), indicating that INFs cultured *in vitro* grew significantly faster compared with NFs. However, the apoptotic rates of INFs (6.12% ± 0.12%) and NFs (6.25% ± 0.14%) are identical (P > 0.05), as analyzed by FITC-Annexin V/PI staining and flow cytometry (Fig. 2C).

In addition, fibroblast activation protein (FAP) and alpha-smooth muscle actin (α-SMA), two indicators of myofibroblasts, exhibited increased protein expression in INFs compared with NFs (Fig. 2D).

Furthermore, given that tumor necrosis factor-α (TNF-α) and interleukin (IL)-6 are well-known inflammatory mediators during mastitis, their secretion in medium by INFs and NFs were examined using ELISA kits. We detected significantly elevated levels of TNF-α and IL6 protein secreted in medium by INFs compared with that secreted by NFs (Fig. 2E), suggesting that mammary stromal fibroblasts contribute to the inflammatory response during mastitis.

### Global gene expression profiles of INFs and NFs analyzed by RNA-Seq (Quantification)

To compare the gene expression profiles between NFs and INFs, we analyzed the global gene expression levels by using Illumina HiSeqTM 2000. Initially, we generated two types of mRNA libraries (two repeats for each library), one from NFs and the other from INFs. The major characteristics of these libraries are summarized in Table 1. We obtained 12,167,245 (99.80% of raw reads) from NFs and 12,165,544 clean reads (99.79% of raw reads) from INFs. Of the total clean reads, 84.25% and 84.90% were mapped to the reference genome and 67.01% and 66.31% to the reference gene for the NFs and INFs, respectively (Table 1). These results suggest that clean reads were well mapped to the genome and gene reference sequences.

**Figure 3:**
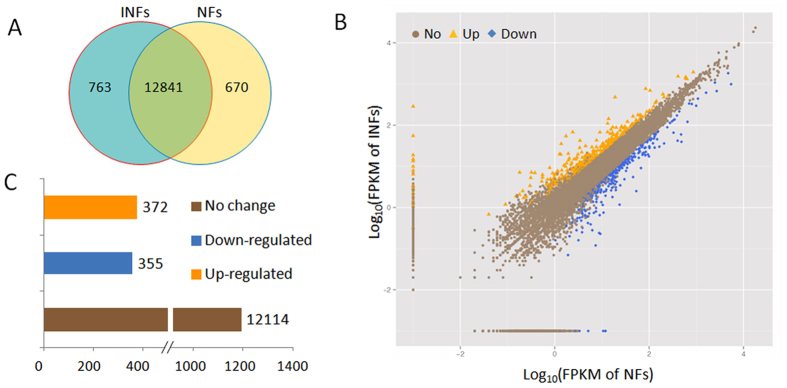
RNA-Seq gene expression results in NFs and INFs. **(A)** Venn diagram showing the number of co-expressed genes and unique genes between NFs and INFs. **(B)** Scatter plot of co-expressed genes between NFs and INFs. Blue denotes downregulated genes, orange denotes upregulated genes, and brown denotes non-regulated genes in INFs compared with NFs under the criteria of a FDR < 0.001 and absolute log_2_ (Y/X) > 1. **(C)** Histogram showing the number of differentially expressed genes and non-regulated genes between INFs compared with NFs.

There are a total of 12,841 co-expressed genes between NFs and INFs, 670 unique genes in NFs, and 763 unique genes in INFs (Fig. 3A). Poisson distribution analysis was applied to screen the differential expressed genes (DEGs) from the co-expressed genes between NFs and INFs and conduct a further function analysis on them. Under the criteria of FDR ≤ 0.001 and absolute log_2_ ratio >1, comparison of NFs and INFs showed that 727 genes were significantly differentially expressed. Of the 727 DEGs, 372 and 355 were upregulated and downregulated, respectively, in INFs compared with NFs (Fig. 3B,C).

### Gene ontology (GO) and pathway enrichment analysis of DEGs

The analysis of GO functional enrichment revealed the DEGs were significantly enriched for 10 functional terms. Among these, four terms were enriched from the cellular component ontology, one term from the molecular function ontology, and five terms from the biological process ontology (Table 2).

**Figure 4:**
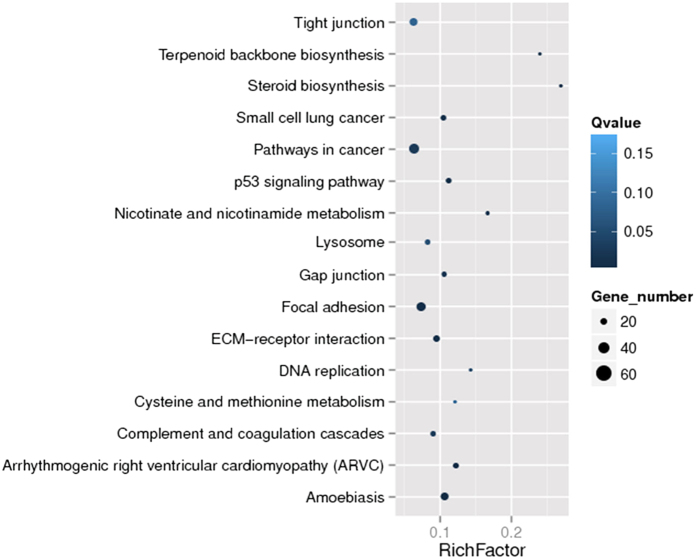
Scatter plot for KEGG enrichment results. The Rich factor is the ratio of differentially expressed gene numbers annotated in this pathway term to all gene numbers annotated in this pathway term. The greater the Rich factor, the greater the degree of pathway enrichment. A Q value is the corrected p value ranging from 0 to 1, and a lower value indicates greater pathway enrichment.

The pathway enrichment analysis revealed that, of all genes with a Kyoto Encyclopedia of Genes and Genomes (KEGG) annotation, 618 DEGs with pathway annotation were assigned to 228 KEGG pathways (Table S1). We filtered the pathways that were significantly enriched in DEGs with Q value ≤0.05. Fourteen of these 228 KEGG pathways were significantly enriched. We generated a scatter plot for these enriched results, as shown in Fig. 4. Two additional pathways with Q value = 0.07 were also included in the scatter plot owing to similarity of functions with the significant enriched pathways. The enriched pathways could be grossly divided into three functional aspects, covering cell junction and adhesion, immune response, as well as biosynthesis and metabolism. Pathways related to cell junction and adhesion included focal adhesion, gap junction, tight junction, adherens junction, regulation of actin cytoskeleton, and vascular smooth muscle contraction. Immune response-related pathways included amoebiasis, extracellular matrix (ECM)-receptor interaction, the TGF-beta signaling pathway, and phagosomes. Others were mainly linked to biosynthesis and metabolism, such as steroid biosynthesis, terpenoid backbone biosynthesis, as well as nicotinate and nicotinamide metabolism.

### DEGs related to ECM remodeling, cell junction and focal adhesion, and immune response between INFs and NFs

On the basis of the data obtained using the Go Function analysis and the Pathway Enrichment analysis, we conducted an in-depth analysis of the DEGs between two types of fibroblasts. We listed three groups of DEGs that are respectively related to ECM remodeling, cell junction and focal adhesion, and immune response.

**Figure 5:**
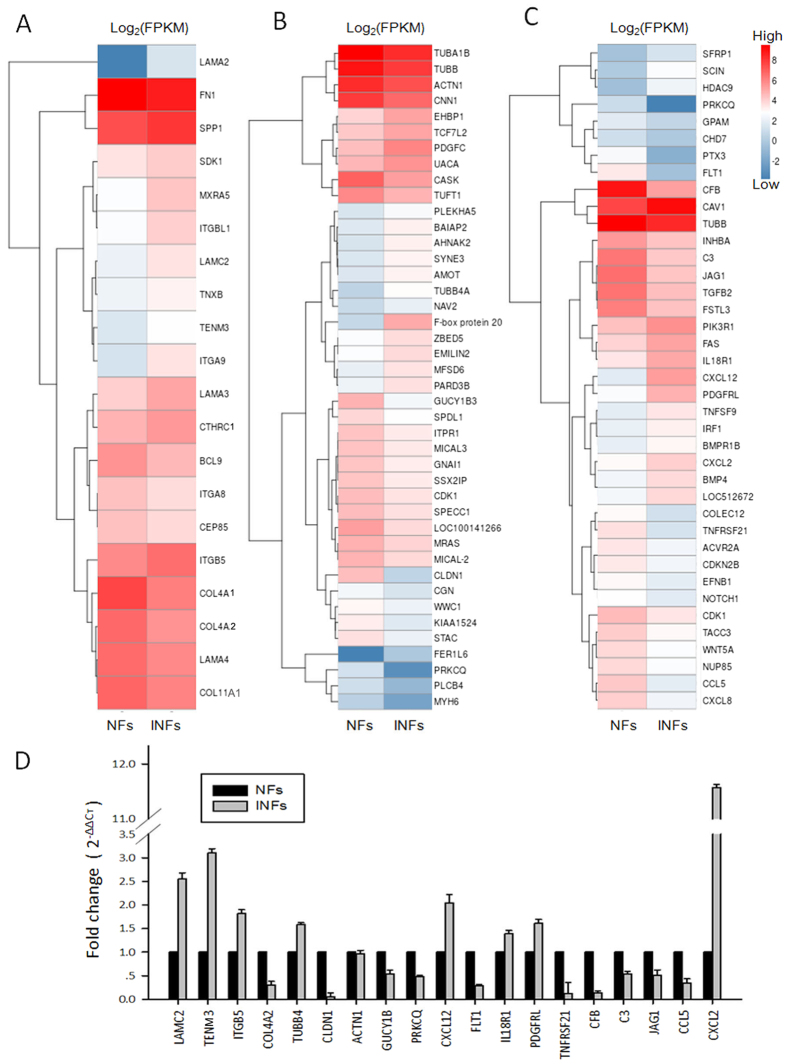
Cluster analysis of differential gene expression pattern. The heatmaps show the expression levels of 20 ECM remodeling-related differentially expressed genes (DEGs) **(A)**, 42 cell junction- and focal adhesion-related DEGs **(B)**, and 39 immune response-related DEGs **(C)**. Nineteen DEGs were randomly selected, and their expression levels were further confirmed by real-time PCR. Each sample was measured in triplicate and the data were representative for one of three independently repeated experiments **(D)**.

Fibroblasts are primarily responsible for the synthesis and remodeling of ECM in tissues during tissue development, differentiation, and repair in many organs^22^. A list of 20 genes related to ECM remodeling was provided in Table S2, and a heat map of these genes was presented (Fig. 5A). Among the 20 genes, the gene expression levels of laminin alpha 2 (LAMA2), laminin gamma 2(LAMC2), integrin beta-like 1 (ITGBL1), and Integrin alpha 9 (ITGA9) were markedly upregulated, whereas the type IV collagen and alpha 1 (COL4A1) were significantly downregulated in INFs compared with NFs (absolute log_2_ ratio >  2).

Increasing evidence suggests that the fibroblast should be considered a sentinel cell on the basis of the ability of the fibroblast to function both as a structural element and as a vital immunoregulatory cell^9^. We also screened 42 of DEGs related to cell junctions or focal adhesion between NFs and INFs, listed in Table S3 and mapped in Fig. 5B. Of these genes, fer-1-like family member 6 (FER1L6), tubulin beta 4A class Iva (TUBB4A), AHNAK nucleoprotein 2 (AHNAK2), and F-box protein 20 (Fbp20) were significantly upregulated; soluble guanylate cyclase 1 beta 3 (GUCY1B3), phospholipase C beta 4 (PLCB4), protein kinase C theta (PRKCQ), myosin, heavy chain 6 (MYH6), and claudin 1 (CLDN1) were markedly downregulated in INFs compared with NFs(absolute log_2_ ratio > 2). In addition to the DEGs linked to structural elements, 39 immune-related genes were differentially expressed in NFs and INFs, and most of these genes were chemokines, cytokines, growth factors, or their corresponding receptors, such as chemokine (C-X-C motif) ligand 12 (CXCL12), chemokine (C-C motif) ligand 5 (CCL5), complement factor B (CFB), and the Fas cell surface death receptor (FAS). (Table S4 and Fig. 5C).

To confirm the RNA-Seq results, we performed real-time PCR with more than a dozen of randomly selected genes, and the mRNA libraries constructed for RNA-Seq were used for confirmation. We found that the expression of these selected genes was consistent with the results, as determined by RNA-Seq (Fig. 5D). In addition, the expression levels of these selected genes in each individual were also examined by real-time PCR to check the variations among the samples used for constructing the library. The data showed that the expression levels of all selected genes match the results of RNA-Seq (Fig. S1).

### Effects of INFs on the characteristics and functions of epithelial cells

**Figure 6:**
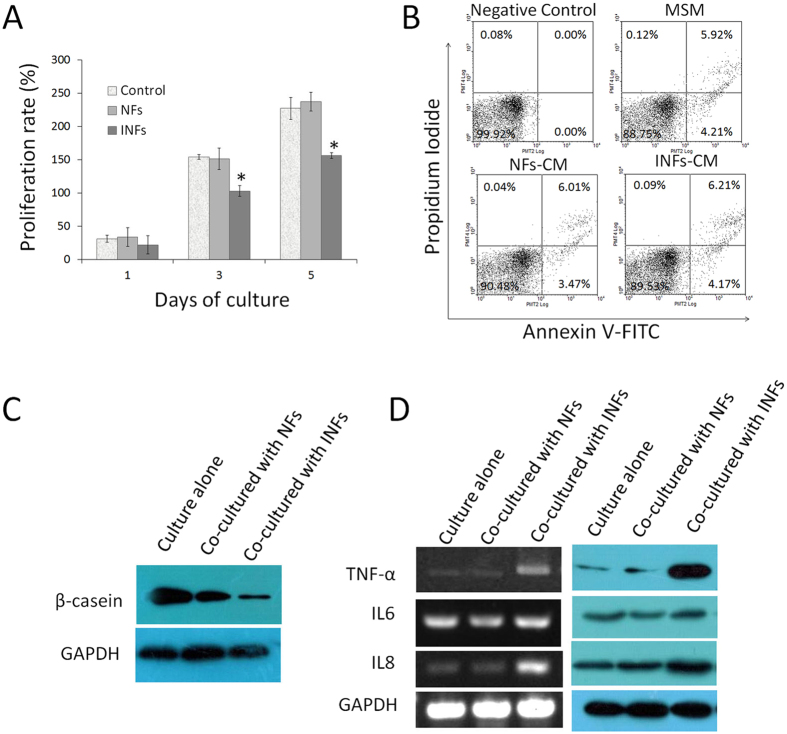
Effects of INFs on proliferation, apoptosis, and inflammation-related factor expression of epithelial cells. **(A)** INFs inhibited the proliferation of epithelial cells compared with NFs in an indirect co-culture model. Minimum serum medium (MSM) composed of DMEM/F12, 0.5% FBS, and antibiotics was used as control. *P < 0.01 versus NFs and control. The data were derived from three independent experiments. **(B)** Epithelial cells exhibited identical apoptotic rates when they were cultured in INFs-conditioned medium (CM), NFs-CM, and MSM. The percentages of apoptotic cells were plotted, and the data were derived from a representative experiment performed in triplicate. **(C)** The β-casein expression was detected by Western blot analysis. Epithelial cells co-cultured with INFs expressed lower level of β-casein compared with those co-cultured with NFs. **(D)** The expression levels of TNF-α, IL6, and IL8 were detected by Reverse transcription PCR (left panel) and Western blot analysis (right panel). The results showed that INFs upregulated the expression of interleukin 8 (IL8) and tumor necrosis factor- alpha (TNF-α) but did not affect the expression of IL6 in epithelial cells. The pooled cells were measured in triplicate and all data were representative for one of the three independently repeated experiments. All representative images were obtained from one of three independently repeated experiments.

To further investigate whether the fibroblasts extracted from infected mammary gland affect the characteristics and functions of epithelial cells that were not yet inflamed in the bovine mammary tissue with mastitis, normal epithelial cells extracted from a normal mammary tissue were co-cultured with INFs and NFs, respectively, *in vitro* in an indirect co-culture model by using conditioned medium (CM). The effects of INFs on the proliferation and apoptosis of epithelial cells were evaluated. The proliferation rate of epithelial cells cultured in INFs-CM was significantly lower than those of epithelial cells cultured in NFs-CM and control medium on Days 3 and 5; no difference was observed among each group on Day 1 (Fig. 6A). In addition, FITC-AnnexinV/PI staining and flow cytometry indicated that the apoptotic rates of the epithelial cells were identical (P > 0.05) regardless of whether they were co-cultured with NFs or INFs (Fig. 6B). The apoptotic rates were (9.37 ± 2.27) % and (9.21 ± 2.05) %, respectively.

The hallmark of mammary epithelial cells is copious secretion of milk-specific components, and β-casein is a major protein component of milk. Epithelial cells were co-cultured in 6-well plates by using cell culture inserts, and β-casein secretion was measured using an ELISA kit to address the effects of INFs on the β-casein secretion of epithelial cells. We detected a lower (p < 0.01) amount of β-casein secreted in the medium in which epithelial cells were co-cultured with INFs (16.5 ± 0.3) ng/L compared with NFs (19.7 ± 0.5) ng/L. β-casein protein expression was also lower in the epithelial cells co-cultured with INFs compared with those co-cultured with NFs (Fig. 6C).

Mammary epithelial cells are highly immune-competent except for the resident population of immune cells; elevated mRNA concentrations for key cytokines of TNF-α, IL-1, and IL-8 have been observed in epithelial cells with infection^23^. In the present study, we found that INFs upregulated the expression of TNF-α and IL8 in epithelial cells but did not affect the expression of IL6 at both mRNA and protein levels in an indirect co-culture model by using cell culture inserts (Fig. 6D).

## Discussion

Although considerable advances have been made in understanding the pathobiology of mastitis, important areas remain poorly understood. Previous studies have shown that cellular components of neutrophils^15^, macrophages^16^,^17^, and lymphocytes^18^, as well as epithelial cells in mammary gland^24^ mediate the immune response during mastitis. In addition to these cellular components, our results show that stromal fibroblasts derived from bovine mammary gland with mastitis are also crucial inflammatory cells and involved in the disease process. Our conclusion is based on the observation that mammary stromal fibroblasts derived from mastitic cows show inflammation-specific changes compared with those from cows without mastitis, including the following: relatively abundant lysosomal vacuoles; enhanced activities as indicated by FAP and α-SMA expression; increased proliferation capacity; elevated TGF-α and IL6 secretion; upregulated inflammation-related genes; as well as inhibitory effects on the proliferation and β-casein secretion of epithelial cells and stimulatory effects on the TGF-α and IL6 expression in epithelial cells.

The involvement of innate and adaptive immune cells in bovine mastitis has been well established^18^,^19^,^25^; however, few studies have implicated fibroblasts in this process. Fibroblasts have been shown to participate in the pathogenesis of chronic inflammatory diseases^23^, such as rheumatoid arthritis and chronic obstructive pulmonary disease, as well as allergic inflammation^26^. Fibroblasts have also been suggested to mediate the transition from acute to chronic inflammation owing to disordered fibroblast behavior in which failure to switch off their inflammatory program leads to the inappropriate survival and retention of leukocytes within the inflamed tissue^20^. Furthermore, fibroblasts can synthesize chemokines to regulate Inflammation as sentinel cells^9^. Extending on these findings, our results unequivocally demonstrate that INFs vary from NFs with respect to basic biological and molecular characteristics and functional contributions to epithelial cells.

Fibroblasts found in different anatomical locations and disease status, even within the same tissue, show differences in proliferation, collagen and matrix metalloproteinase production, and immunomodulatory function; these differences are maintained after an extended *in vitro* culture^27^. INFs and NFs exhibited identical morphologies characterized as long and spindle-shaped under phase contrast microscope. They also showed well-developed rough endoplasmic reticuli, mitochondria, and Golgi apparatus (except for abundant lysosomal vacuoles in INFs under transmission electron microscope); nevertheless, INFs and NFs could still be distinguished based on the differences in growth rate, FAP and α-SMA expression, as well as TNF-α and IL6 secretion. Oda *et al.* suggested that both proliferation and apoptosis should be considered in predicting cell growth^28^. Our data indicated that the higher proliferation rate of INFs than that of NFs was not accompanied by either higher or lower apoptosis, suggesting that this more rapid growth rate of INFs was mainly determined by the rapid proliferation rate. Activated fibroblasts found in damaged and inflamed tissues or tumor stroma are able to express high α-SMA^29^ and FAP^30^. Consistent with these findings, we found that INFs exhibited a higher expression of FAP and α-SMA compared with NFs, indicating that fibroblasts were active within the mammary gland during mastitis^31^. We also found that INFs could secrete elevated cytokines of TNF-α and IL6 compared with NFs. Cytokines IL-6 and TNF-α are considered important to elicit the acute phase response and allow the accumulation of leukocytes at the site of infection^19^. Likewise, epithelial can reportedly release neutrophil-mobilizing chemokines and pro-inflammatory cytokines upon bacterial stimulation during mastitis^31^. We may infer that increased cytokines of IL-6 and TNF-α secreted by activated fibroblasts within mammary stroma during mastitis could cooperate with epithelial cells, recruit leukocytes to alveoli, and promote inflammation. This inference agrees with the concept that fibroblasts provide survival and retention signals for leukocytes leading to their inappropriate and persistent accumulation within inflamed tissues^10^.

The aforementioned differences between INFs and NFs are corroborated by comparative transcriptome analysis, which revealed different transcriptional profiles for the two types of fibroblasts. In the current study, only the DEGs between NFs and INFs were analyzed because only few unique genes expressed by NFs or INFs were enriched when GO and KEGG pathway analysis were performed. The functions and pathways that the enriched unique genes in NFs and INFs belong to are provided in Tables S5–S7.

Mastitis could cause tissue damage of the mammary gland, and the damaged secretory tissue is replaced with a nonsecretory tissue consisting predominantly of fibroblasts^1^. Fibroblasts are primarily responsible for the synthesis and remodeling of ECM in tissues and play a critical role during tissue development, differentiation, and repair in many organs^22^. Our data indicated that 20 DEGs are related to ECM remodeling, such as LAMA2, LAMA3, LAMC2, and collagen triple helix repeat-containing protein 1(CTHRC1) (Table S2). Fragments of collagen and laminin have been reported to promote chemotaxis of monocytes and neutrophils within the interstitial tissue^32^, and one of the characteristics of mastitis is the influx of somatic cells, primarily polymorphonuclear neutrophils, into the mammary gland. Chemokines and (or) cytokines secreted by epithelial cells in response to pathogens are known to facilitate neutrophil infiltration into alveolars. In the present study, we suggest that fibroblasts also mediate, at least partly, the recruitments of neutrophils during mastitis given that INFs exhibited a higher expression of certain collagens and laminins.

Smith *et al.* reviewed that fibroblasts can function both as structural elements and as vital immunoregulatory cells^9^. We observed 42 DEGs related to cell junctions or focal adhesion, for instance, several members of the tubulin family, claudin 1, actinin-α 1, and myosin (Table S3) and 39 immune-related DEGs, including CXCL2, CXCL12, and the TNF receptor superfamily member 21 (Table S4). The downregulation of tubulin, claudin 1, and actinin-α 1 may damage the junction among fibroblasts, which may promote pathogens to invade the surrounding stroma of the mammary gland after breaking the basement membrane and epithelium of alveoli. The invasion of pathogens into the stroma and their products may increase the severity of the inflammation. CXCL12 (also named as SDF1) is a new chemokine that is chemotactic to neutrophils^33^. The elevated inflammatory mediators that we found in this study, including the CXCL12 secreted by fibroblasts, may recruit neutrophils from blood into the alveolus in cooperation with inflammatory mediators secreted by other cells within the mammary gland during mastitis. We did not address the specific functions of each gene during mastitis; however, we can conclude that fibroblasts isolated from the mammary gland of mastitic cows have different gene expression profiles compared with those from cows without mammary gland infection.

Our study also demonstrated that compared with NFs, INFs inhibited normal epithelial cell proliferation and reduced the secretion of β-casein, a major protein component of milk, in a co-culture model. In addition, INFs inhibited normal epithelial cell proliferation compared with NFs and the control medium. Furthermore, INFs induced the inflammatory reaction of normal epithelial cells, as indicated by elevated TNF-α and IL8 expression. The findings in this *in vitro* co-culture model suggest that the activated fibroblasts can result in the spread of inflammation to the uninflamed epithelial cells in the alveolus during mastitis, followed by deceased lactation.

In summary, these variations in phenotype and function that characterize fibroblasts derived from the mammary glands of cows with or without mastitis suggest that fibroblasts play significant roles during mastitis. Ignoring the contribution of fibroblasts to the pathogenesis of bovine mastitis may account for the failure of current therapies to provide a permanent cure. We suggest that, in addition to epithelial cells or other cell types in the mammary glands, fibroblasts can be studied further as targets for the treatment of mastitis. A better understanding of the interplay between epithelial cells and different stroma-derived cell types within the mammary gland, including neutrophils, macrophages, lymphocytes, as well as fibroblasts, are important in developing strategies for improved bovine mastitis therapy.

## Materials and Methods

### Isolation of bovine mammary stromal fibroblasts and epithelial cells

Mammary tissues were obtained from Holstein dairy cows introduced to the slaughterhouse because of problems of clinical mastitis or fractured legs during middle or late lactation. All cows were unrelated, multiparous, and aged from four years to nine years. An experienced anatomical pathologist conducted a gross examination and obtained representative samples of tissue. A fraction of each tissue sample was fixed in formalin and embedded in paraffin for routine histopathological examination. The remainder was used to isolate primary fibroblasts and epithelial cells in accordance with a previously described procedure^34^. Fresh tissues were cut into smaller pieces, washed with phosphate buffer saline, and placed in a digestion solution of Enzyme Cocktail (ISU ABXIS, Seoul, Korea) and incubated at 37 °C overnight in a humidified 5% CO_2_ incubator. Digested tissue was filtered through a 70 μm cell strainer. The preparation was centrifuged to yield a pellet containing epithelial organoids, undigested tissue fragments, and a supernatant containing fibroblasts. The isolated epithelial cells and fibroblasts were cultured in DMEM/F12 supplemented with 10% fetal bovine serum (FBS) and 100 IU/ml penicillin with 100 μg/ml streptomycin (Gibco BRL, Grand island, NY), respectively, and cultured at 37 °C in a humidified 5% CO_2 _environment. Morphological and immunohistochemical characterization of cells were conducted using anti-wide spectrum cytokeratin (ab9377, diluted 1:500, Abcam, Cambridge, UK) and anti-vimentin (ab8978, diluted 1:500, Abcam). Fibroblasts and epithelial cells were used within the fifth passage.

Bacterial contamination of all obtained tissue categorized as inflamed (presumptively infected) and normal (presumptively uninfected) tissue was determined by culturing 10 μl of residual milk from mammary tissue samples on blood agar plates by using standard bacteriological techniques. The degree of bacterial contamination was evaluated after cultivation on blood agar for 24 h at 37 °C and categorized as uninfected if no colonies were formed. The plates that resulted in at least 1 colony forming unit (CFU) were sent to Microbiology Experimental Center of Yangling Demonstration Zone Hospital (Yangling, Shaanxi, China), where pathogenic bacteria on the plates were identified using additional laboratory tests in accordance with the laboratory standards.

### ELISA

For detection of TNF-α and IL-6 secreted by NFs or INFs, cells were cultured and grown to 80% confluence. The medium was replaced with minimum serum medium (MSM) composed of DMEM/F12, 0.5% FBS, and antibiotics and then cultured for another 48 h. For detection of β-casein secreted by epithelial cells, the cells were co-cultured with INFs or NFs in 6-well plates by using cell culture inserts with a membrane containing 0.4 μm pores. Epithelial cells were seeded in 6-well plates. INFs or NFs were seeded in inserts and cultured for three days in MSM. The media were finally collected and centrifuged to remove the cell debris. The amount of TNF-α, IL-6, and β-casein in the medium was measured with corresponding immunoassay kits (Huzhen Biological Technology, Shanghai, China) in accordance with the manufacturer’s instructions.

### RNA purification, cDNA library construction, and RNA-Seq

Total RNA was respectively extracted from NFs and INFs and purified using Trizol reagent (Invitrogen, Carlsbad, CA) in accordance with the manufacturer’s instructions. The quality and quantity of the purified RNA samples were assessed using Agilent 2100 Bioanalyzer. RNA samples with high purity (28S/18S > 2.0) and high integrity (RIN > 8.0) were used for cDNA library construction. The total RNA samples were first treated with DNase I to degrade any possible DNA contamination and then enriched using oligo(dT) magnetic beads. The mRNA was then fragmented into about 200 bp short fragments and used as template to synthesize the double-strand cDNA by using random hexamer-primer, buffer, dNTPs, RNase H, and DNA polymerase I. The cDNA was then purified with magnetic beads. End reparation and 3′-end single nucleotide A (adenine) addition were performed. Finally, the RNA fragments were ligated using the sequencing adapters and enriched by PCR amplification. During the QC step, Agilent 2100 Bioanalyzer and ABI StepOnePlus Real-Time PCR System were used to qualify and quantify the sample library. The library products were prepared for sequencing using Illumina HiSeq^TM^ 2000 (Beijing Genomics Institute (BGI), Shenzhen, China).

### Data processing and DEG screening

The clean reads were obtained from raw reads after removal of the reads with adapters, reads with more than 10% unknown bases, and reads with more than 50% low-quality bases, in accordance with the criteria of low-quality bases with sequencing quality no higher than 5. All clean reads were then well mapped to the genome and gene reference sequences to calculate the ratio of alignment. In general, the higher ratio of alignment indicates a closer genetic relationship between the samples and the reference species.

The method based on fragments per kilobase of exon per million fragments mapped (FPKM) is used to calculate gene expression, and the equation (1) was used:


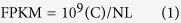


where the given FPKM(A) is the expression of gene A, C is the number of reads uniquely aligned to gene A, N is the total number of reads uniquely aligned to all genes, and L is the number of bases in gene A.

Poisson distribution analysis was performed to screen the DEGs between NFs and INFs according to a previous publication in which the algorithm was provided in detail^35^. We used “FDR ≤ 0.001 and the absolute value of log_2_Ratio ≥1” as the threshold to evaluate the significance of the difference in gene expression^36^.

### GO functional enrichment analysis for DEGs

GO enrichment analysis of functional significance applied a hypergeometric test to map all differentially expressed genes to terms in the GO database, searching for significantly enriched GO terms in DEGs compared with the genomic background and calculated according to the equation (2):


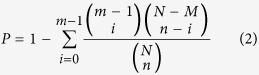


where N is the number of all genes with GO annotation; n is the number of DEGs in N; M is the number of all genes that are annotated to certain GO terms; m is the number of DEGs in M. The calculated p-value was adjusted using the Bonferroni correction, and the corrected p-value ≤ 0.01 was selected as the threshold. GO terms fulfilling this condition are defined as significantly enriched GO terms in DEGs. After obtaining the GO annotation for DEGs, we used the WEGO software^37^ to perform GO functional classification for enriched DEGs.

### Pathway enrichment analysis for DEGs

A pathway analysis for DEGs was performed based on the KEGG database^38^, the major public pathway-related database. This analysis identifies significantly enriched metabolic pathways or signal transduction pathways in DEGs relative to the whole genome background. The formula is the same as that used in GO analysis. N is the number of all genes with KEGG annotation, n is the number of DEGs in N, M is the number of all genes annotated to specific pathways, and m is the number of DEGs in M. Pathways with Q ≤ 0.05 were considered significantly enriched.

### Reverse transcription PCR (RT-PCR) and quantitative real-time PCR analysis

Ribonucleic acid was extracted from cells using Trizol reagent (Invitrogen, Carlsbad, CA) in accordance with the manufacturer’s instructions. Complementary DNA was synthesized using 0.5–2 μg of total RNA with a PrimeScript RT reagent kit (TaKaRa, Dalian, Liaoning, China), and 2 μl of cDNA product was amplified by RT-PCR. GAPDH was used as an internal control in all reactions. PCR products were separated on 2% agarose gel and stained with ethidium bromide. Real-time quantification of mRNA was performed with an ABI StepOnePlus PCR system (Applied Biosystems, Foster City, CA) using SYBR Premix ExTaq II kits (Takara). The comparative Ct method was employed for quantification of target gene expression that was normalized to GAPDH expression and relative to the calibrator. Data were expressed as the fold change (FC) = 2^−ΔΔCt^. A list of all primers used in this study was presented in Table S8.

### Cell proliferation and apoptosis assay

Cell proliferation and apoptosis assays were performed using the Cell Counting Kit-8 (Dojindo, Rockville, MD) and FITC Annexin V Apoptosis Detection Kit I (BD Pharmingen, San Diego, CA). For cell proliferation of NFs and INFs, cells were plated in 96-well plates at the same density of 2,000 cells/well in growth medium and then cultured for indicated days for cell proliferation assay. On Days 0, 1, 3, and 5, the absorbance of samples in triplicate wells was measured with a VersaMax Microplate Reader at a wavelength of 450 nm, and the cell proliferation rates were calculated using the equation (3):


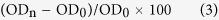


where OD_n_ means optical density value at indicated day after culture. For apoptosis assay of NFs and INFs, cells were respectively cultured in growth medium and grown to confluence; cells were then routinely harvested to analyze apoptotic rates by using the FITC Annexin V/PI Apoptosis Detection Kit in accordance with the manufacturer’s instructions.

For proliferation and apoptosis examination of epithelial cells, cells were cultured for three days in conditioned medium (CM) obtained from NFs or INFs cultured in MSM for three days. The proliferation and apoptosis rates were then analyzed using similar methods as described in previous sections.

### Western blot analysis

Cells were lysed in PRO-PREP Protein Extraction Solution (iNtRON Biotechnology, Inc., Gyeonggi-do, Korea). Total protein ranging from 5 μg to 20 μg from each sample was resolved by SDS-PAGE on a 4–12% Bis-Tris Gel with running buffer and transferred to polyvinylidene difluoride membranes. The blots were then probed with antibodies against FAP (1:1000 dilution, Biosynthesis Biotechnology, Beijing, China), α-SMA (1:1000 dilution, Sigma-Aldrich, St Louis, MO), and GAPDH (1:500 dilution, Santa Cruz Biotechnology, Dallas, TX).

### Statistical analysis

The results are expressed as means ± standard deviation (s.d.). All statistical analyses were performed using ANOVA with the Bonferroni post hoc test. (SPSS 11.5, IBM Corporation, Armonk, NY). P < 0.05 was considered statistically significant.

## Additional Information

**How to cite this article**: Chen, Q. *et al.* Stromal fibroblasts derived from mammary gland of bovine with mastitis display inflammation-specific changes. *Sci. Rep.* **6**, 27462; doi: 10.1038/srep27462 (2016).

## Supplementary Material

Supplementary Figure S1, Table S2-8

Supplementary Dataset 1

## Figures and Tables

**Table 1:** Major characteristics of mRNA libraries and database generated by RNA-Seq.

Summary	Normal	Mastitis
Raw reads containing		
N	1978 (0.02%)	1811 (0.01%)
Adapters	7244 (0.06%)	7696 (0.06%)
low quality	15118 (0.12%)	16384 (0.13%)
clean reads	12167245 (99.80%)	12165544 (99.79%)
Genome map rate of clean reads	84.25%	84.90%
Unique match in genome-mapped reads	77.51%	77.82%
Gene map rate of clean reads	67.01%	66.31%
Unique match in gene-mapped reads	61.27%	60.31%

**Table 2:** GO functional enrichment analysis of DEGs between NFs and INFs.

Gene ontology term	Cluster frequency*	Genome frequency of use	Corrected P-value
Terms from the Component Ontology			
extracellular matrix	22/422	163/10103	0.00013
kinetochore	11/422	47/10103	0.00033
extracellular region	43/422	492/10103	0.00038
chromosome	30/422	323/10103	0.00413
Terms from the Function Ontology			
oxidoreductase activity	4/422	6/9897	0.00945
Terms from the Process Ontology			
cholesterol metabolic process	11/429	33/9543	0.00012
regulation of developmental process	38/429	381/9543	0.00339
negative regulation of developmental process	17/429	109/9543	0.00756
tissue development	39/429	413/9543	0.00937
negative regulation of cellular process	56/429	688/9543	0.00985

^*^Cluster frequency: the denominator represents the total number of genes with GO annotation, and the numerator represents the number of each GO term genes. Genome frequency of use: the denominator represents the number of reference genes with GO annotation, and the numerator represents the number of reference genes annotated in the listed GO term. Corrected P-value: P-value in hypergeometric test after correction.
