# Supplementary material for: Stromal fibroblasts derived from mammary gland of bovine with mastitis display inflammation-specific changes
Source: Sci Rep. 2016 Jun 7;6:27462. doi: 10.1038/srep27462 (PMC4895242; doi:10.1038/srep27462)
Supplement: Supplementary Figure S1, Table S2-8 [file srep27462-s1.pdf]

Stromal fibroblasts derived from mammary gland of bovine with mastitis display inflammation-specific changes

Qing Chen[1※], Guiliang He[1※], Wenyao Zhang[1], Tong Xu[1], Hongliang Qi[1], Jing Li[1], Yong Zhang[1,2,]* & Ming-Qing Gao[1,2,]*

[1]*College of Veterinary Medicine, Northwest A&F University, Yangling 712100, Shaanxi, China*
[2]*Key Laboratory of Animal Biotechnology, Ministry of Agriculture, Northwest A&F University, Yangling 712100, Shaanxi, China*

[※]These authors contributed equally to this work.
*Correspondence and requests for materials should be addressed to M.-Q.G. (email: gaomingqing@nwsuaf.edu.cn) or Y.Z. (email: zhangyong1956@nwsuaf.edu.cn)

Fig S1.

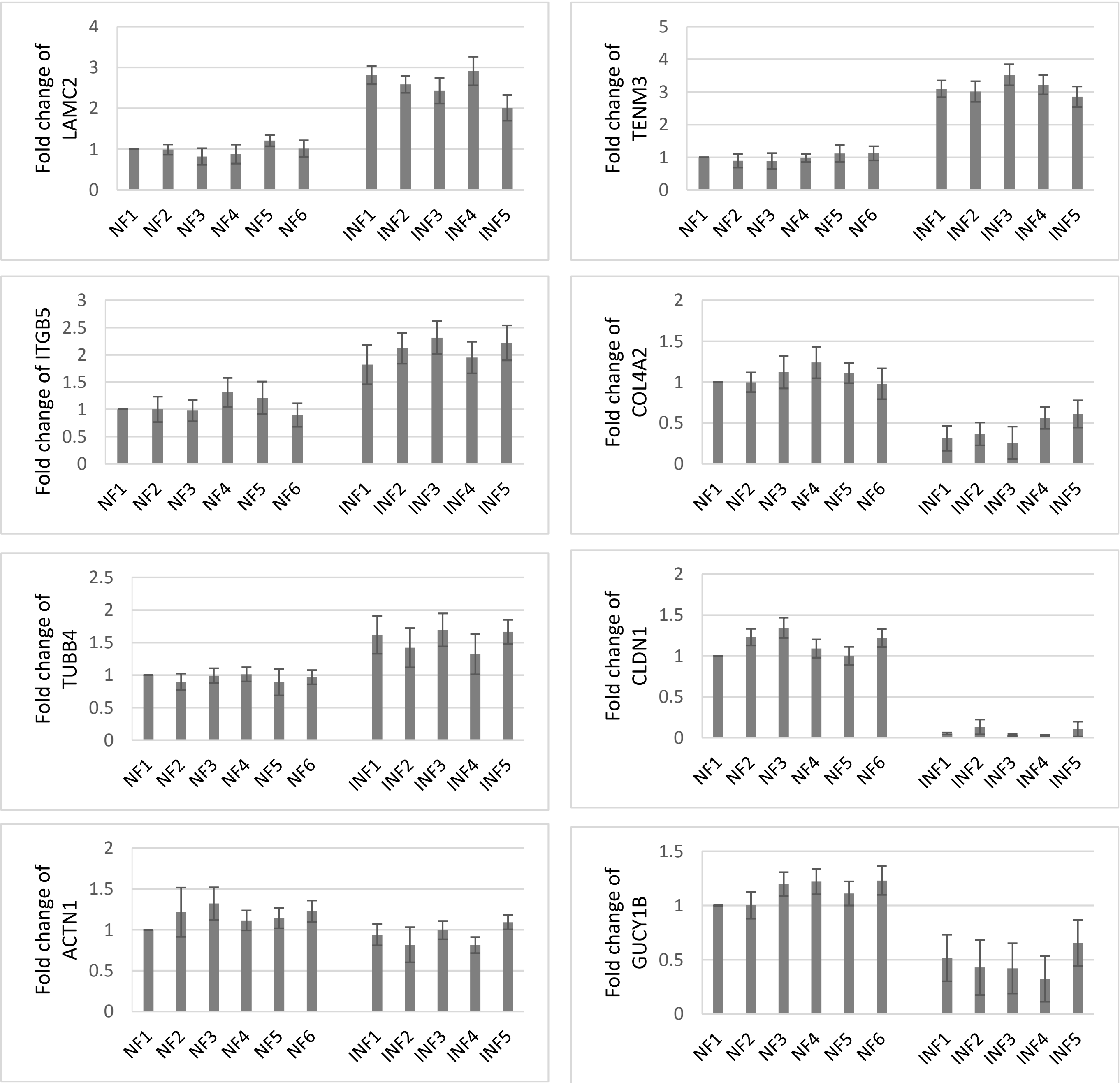

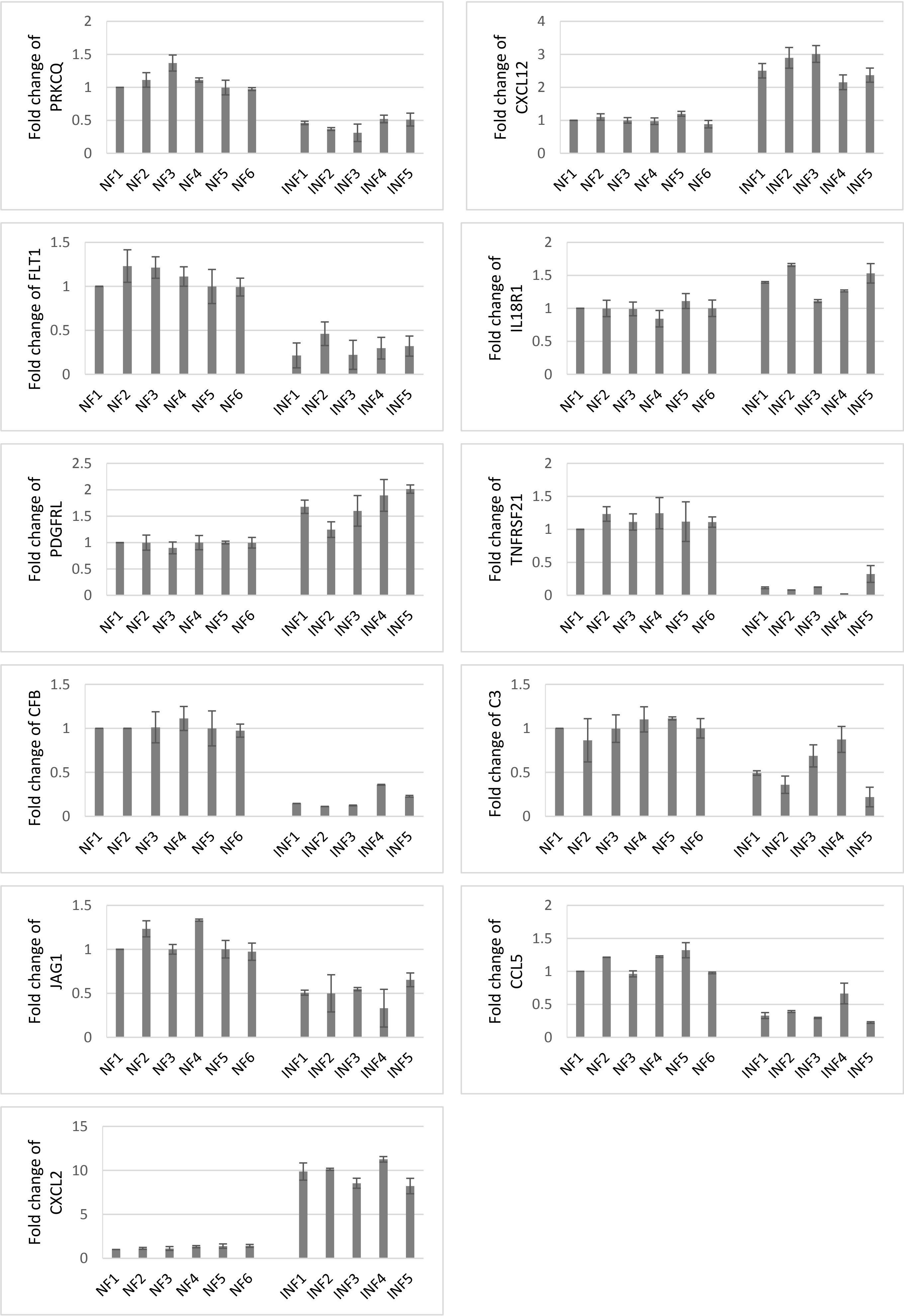
Fold change of PRKCQ
2
1.5
1
0.5
0
NF1 NF2 NF3 NF4 NF5 NF6 INF1 INF2 INF3 INF4 INF5
Fold change of CXCL12
4
3
2
1
0
NF1 NF2 NF3 NF4 NF5 NF6 INF1 INF2 INF3 INF4 INF5
Fold change of FLT1
1.5
1
0.5
0
NF1 NF2 NF3 NF4 NF5 NF6 INF1 INF2 INF3 INF4 INF5
Fold change of IL18R1
2
1.5
1
0.5
0
NF1 NF2 NF3 NF4 NF5 NF6 INF1 INF2 INF3 INF4 INF5
Fold change of PDGFRL
2.5
2
1.5
1
0.5
0
NF1 NF2 NF3 NF4 NF5 NF6 INF1 INF2 INF3 INF4 INF5
Fold change of TNFRSF21
2
1.5
1
0.5
0
NF1 NF2 NF3 NF4 NF5 NF6 INF1 INF2 INF3 INF4 INF5
Fold change of CFB
1.5
1
0.5
0
NF1 NF2 NF3 NF4 NF5 NF6 INF1 INF2 INF3 INF4 INF5
Fold change of C3
1.5
1
0.5
0
NF1 NF2 NF3 NF4 NF5 NF6 INF1 INF2 INF3 INF4 INF5
Fold change of JAG1
1.5
1
0.5
0
NF1 NF2 NF3 NF4 NF5 NF6 INF1 INF2 INF3 INF4 INF5
Fold change of CCL5
2
1.5
1
0.5
0
NF1 NF2 NF3 NF4 NF5 NF6 INF1 INF2 INF3 INF4 INF5
Fold change of CXCL2
15
10
5
0
NF1 NF2 NF3 NF4 NF5 NF6 INF1 INF2 INF3 INF4 INF5

Fig S1. The expressions of the selected genes in the fibroblasts extracted from each individual were confirmed by real time PCR. NF1~NF6 and INF1~INF5 refer to the fibroblasts from each individual before mixture into cell pools. The gene expression was normalized to GAPDH and relative to NF1. The expressions of these selected genes were consistent with the results as determined by RNA-Seq. Each sample was measured in triplicate and the data were representative for one of three independently repeated experiments.

Table S2. List of ECM remodeling-related DEGs in INFs and NFs

gene	gene symbol	log_2ratio
Laminin, alpha 2	LAMA2	8.3
Laminin, gamma 2	LAMC2	2.2
Laminin, alpha 3	LAMA3	1.7
Laminin, alpha 4	LAMA4	-1.3
Collagen triple helix repeat-containing protein 1	CTHRC1	1.2
Collagen, type IV, alpha 1	COL4A1	-2.3
B-cell CLL/lymphoma 9	BCL9	-1.5
Collagen, type IV, alpha 2	COL4A2	-1.7
Collagen, type XI, alpha 1	COL11A1	-1.3
Fibronectin 1	FN1	-1.2
Secreted phosphoprotein 1	SPP1	1.0
Teneurin transmembrane protein 3	TENM3	2.0
Tenascin XB	TNXB	1.5
Sidekick cell adhesion molecule 1	SDK1	1.0
Matrix-remodelling associated 5	MXRA5	2.5
Integrin, alpha 8	ITGA8	-1.2
Integrin, alpha 9	ITGA9	3.5
Integrin, beta-like 1 (with EGF-like repeat domains)	ITGBL1	2.2
Integrin, beta 5	ITGB5	1.2
Centrosomal protein 85kda	CEP85	-1.0

Table S3. List of DEGs related cell junction and focal adhesion in INFs and NFs

gene	gene symbol	log_2ratio
Fer-1-like family member 6	FER1L6	4.1
Tubulin, beta 4A class Iva	TUBB4A	2.7
AHNAK nucleoprotein 2	AHNAK2	2.0
Major facilitator superfamily domain containing 6	MFSD6	1.7
Platelet derived growth factor C	PDGFC	1.6
Guanylate cyclase 1, soluble, beta 3	GUCY1B3	-2.6
Phospholipase C, beta 4	PLCB4	-2.3
Tubulin alpha-1C chain-like	LOC100141266	-1.7
Tubulin, alpha 1b	TUBA1B	-1.3
Cyclin-dependent kinase 1	CDK1	-1.2
Inositol 1,4,5-trisphosphate receptor, type 1	ITPR1	-1.1
Tubulin, beta class I	TUBB	-1.1
Guanine nucleotide binding protein (G protein), alpha inhibiting activity polypeptide 1	GNAI1	-1.1

Spectrin repeat containing, nuclear envelope family member 3	SYNE3	1.8
Angiomotin	AMOT	1.7
Par-3 family cell polarity regulator beta	PARD3B	1.6
EH domain binding protein 1	EHBP1	1.4
Neuron navigator 2	NAV2	1.3
Zinc finger, BED-type containing 5	ZBED5	1.2
Elastin microfibril interfacer 2	EMILIN2	1.2
Uveal autoantigen with coiled-coil domains and ankyrin repeats	UACA	1.1
Protein kinase C, theta	PRKCQ	-4.8
Claudin 1	CLDN1	-4.3
myosin, heavy chain 6, cardiac muscle, alpha	MYH6	-2.7
KIAA1524 ortholog	KIAA1524	-1.8
Calcium/calmodulin-dependent serine protein kinase (MAGUK family)	CASK	-1.7
SH3 and cysteine rich domain	STAC	-1.6
Tuftelin 1	TUFT1	-1.3
Spindle apparatus coiled-coil protein 1	SPDL1	-1.1
Cingulin	CGN	-1.1
Muscle RAS oncogene homolog	MRAS	-1.1
Microtubule associated monooxygenase, calponin and LIM domain containing 3	MICAL3	-1.1
Sperm antigen with calponin homology and coiled-coil domains 1	SPECC1	-1.0
Microtubule associated monooxygenase, calponin and LIM domain containing 2	MICAL-2	-1.0
Actinin, alpha 1	ACTN1	-1.0
WW and C2 domain containing 1	WWC1	-1.0
F-box protein 20	Fbp20	4.7
BAI1-associated protein 2	BAIAP2	1.7
Pleckstrin homology domain containing, family A member 5	PLEKHA5	1.2
Transcription factor 7-like 2 (T-cell specific, HMG-box)	TCF7L2	1.0
Calponin 1, basic, smooth muscle	CNN1	-1.3
Synovial sarcoma, X breakpoint 2 interacting protein	SSX2IP	-1.0

Table S4. List of immune-related DEGs in INFs and NFs

gene	gene symbol	log_2ratio
Phosphoinositide-3-kinase, regulatory subunit 1 (alpha)	PIK3R1	1.3
Inhibin, beta A	INHBA	-1.1
Transforming growth factor, beta 2	TGFB2	-1.9
Secreted frizzled-related protein 1	SFRP1	1.7
Follistatin-like 3 (secreted glycoprotein)	FSTL3	-1.8
Wingless-type MMTV integration site family, member 5A	WNT5A	-1.1
Protein kinase C, theta	PRKCQ	-4.8
Collectin sub-family member 12	COLEC12	-1.8
Complement factor B	CFB	-3.6
Caveolin 1, caveolae protein, 22kda	CAV1	1.5
Glycerol-3-phosphate acyltransferase, mitochondrial	GPAM	-1.1
Activin A receptor, type IIA	ACVR2A	-1.1

Complement component 3	C3	-2.2
Cyclin-dependent kinase 1	CDK1	-1.2
Scinderin	SCIN	2.7
Fas cell surface death receptor	FAS	1.3
Pentraxin 3, long	PTX3	-3.9
Tubulin, beta class I	TUBB	-1.1
Interferon regulatory factor 1	IRF1	1.1
Cyclin-dependent kinase inhibitor 2B (p15, inhibits CDK4)	CDKN2B	-1.0
Chromodomain helicase DNA binding protein 7	CHD7	-1.1
Jagged 1	JAG1	-2.3
Bone morphogenetic protein 4	BMP4	1.4
Transforming, acidic coiled-coil containing protein 3	TACC3	-1.2
Histone deacetylase 9	HDAC9	2.8
Ephrin-B1	EFNB1	-1.1
Chemokine (C-X-C motif) ligand 2	CXCL2	1.1
Chemokine (C-C motif) ligand 5	CCL5	-2.4
Description: chemokine (C-X-C motif) ligand 12	CXCL12	3.6
Nucleoporin 85kda	NUP85	-1.1
Chemokine (C-X-C motif) ligand 8	CXCL8	-1.8
Major histocompatibility complex, class I	LOC512672	1.4
Description: notch 1	NOTCH1	-1.1
Tumor necrosis factor (ligand) superfamily, member 9	TNFSF9	1.6
Platelet-derived growth factor receptor-like	PDGFRL	2.3
Fms-related tyrosine kinase 1	FLT1	-3.8
Tumor necrosis factor receptor superfamily, member 21	TNFRSF21	-2.3
Bone morphogenetic protein receptor, type IB	BMPR1B	-1.1
Interleukin 18 receptor 1	IL18R1	-1.6

Table S5. GO functional enrichment analysis of unique genes in INFs

Gene Ontology term	Cluster frequency*	Genome frequency of use	Corrected P-value
**Terms from the Component Ontology**			
ribonucleoprotein granule	3/339	5/10103	0.04878
cytoplasmic ribonucleoprotein granule	3/339	5/10103	0.04878
P granule	3/339	5/10103	0.04878
pole plasm	3/339	5/10103	0.04878
germ plasm	3/339	5/10103	0.04878
**Terms from the Function Ontology**			
None	4 /422	6 /9897	0.00945
**Terms from the Process Ontology**			
DNA recombination	18 /338	132/9543	0.00067

*Cluster frequency: the denominator represents the total number of genes with GO annotation and the numerator represents the number of each GO term genes. Genome frequency of use: the denominator represents the number of reference genes with GO annotation and the numerator represents the number of reference genes annotated in the listed GO term. Corrected P-value: P-value in hypergeometric test after correction. None: no gene enriched for the terms under the criteria of corrected p-value≤0.05.

Table S6. GO functional enrichment analysis of unique genes in NFs

Gene Ontology term	Cluster frequency*	Genome frequency of use	Corrected P-value
**Terms from the Component Ontology**			
None			
**Terms from the Function Ontology**			
G-protein coupled peptide receptor activity	10 /311	82/ 9897	0.04070
**Terms from the Process Ontology**			
None			

*Cluster frequency: the denominator represents the total number of genes with GO annotation and the numerator represents the number of each GO term genes. Genome frequency of use: the denominator represents the number of reference genes with GO annotation and the numerator represents the number of reference genes annotated in the listed GO term. Corrected P-value: P-value in hypergeometric test after correction. None: no gene enriched for the terms under the criteria of corrected p-value≤0.05.

Table S7. The enriched pathways of unique genes in NFs and INFs

Group	Pathway	DEGs with pathway annotation	P value	Q value*	Pathway ID
NFs	Lysine degradation	18 (3.27%)	3.87E-05	0.004687	ko00310
	Ribosome	26 (4.72%)	4.34E-05	0.004687	ko00310
INFs	Lysine degradation	19 (3.08%)	5.20E-05	0.01122679	ko00310

* Pathways with a Q value≤0.05 were considered significantly enriched.

Table S8. A list of all primers used in this study

Target	Sequence(5'-3')	
LAMC2	F:5'-ATCACAGCTCCCTTGATGCC-3'	R:5'-TCGAGGTACCCAGTGCTGTA-3'
TENM3	F:5'-TGTGACCCTAATTGGACTGGC-3'	R:5'-TCCAAATAGTGAGCGATGGTGC-3'
ITGB5	F:5'-CGTACAACAGCATCCGGTCT-3'	R:5'-TAGGATACACCGTCCTGGCA-3'
COL4A1	F:5'-CAGGTCCCAAAGGTTTCCCA-3'	R:5'-GATTCCATTTGTGTGGCCCG-3'
TUBB4	F:5'-CATTGATCCCACGGGCACATA-3'	R:5'-CTCCAGACTGGCCAAACACAA-3'
CLDN1	F:5'-CCGGTCAATGCCAGGTATGAA-3'	R:5'-GTCAGCTTTCGCTTCTGTGTC-3'
ACTN1	F:5'-CCACTTTGACCGGGATCACT-3'	R:5'-CGGCCAGGATCTTGAAGGAA-3'
GUCY1B1	F:5'-GGCGCTGACACCATGTAT-3'	R:5'-CAGCGTTGAGATTGAGGACC-3'
PRKCQ	F:5'-CACGTTCCAGACCAAGGAGA-3'	R:5'-CCTTGCACATCCCGAAGTCT-3'
CXCL12	F:5'-CTCCCTTCAGATCGTGGCAA-3'	R:5'-CCGCACCTTTGCCCTTTTATC-3'
FLT1	F:5'-TCATTAGAGATCAGGAAGCACCC-3'	R:5'-GCTTTGCAGTGATACACGCC-3'
IL18R1	F:5'-CCCCTTCTGAAGAGAAGCCA-3'	R:5'-TCAGGTCAACAGGTCCATGC-3'
PDGFRL	F:5'-AAGCCCTGGAGGATGTTACTG-3'	R:5'-CTTTGGCGTTGGATCAGCAG-3'
TNFRSF21	F:5'-GGCTTCTTCGTGGATGAGTCT-3'	R:5'-GGTTCAGGATGTGGAGCATGT-3'
CFB	F:5'-CGCTTCATTCAAGTTGGCGT-3'	R:5'-TTGGTCTCATGCCCCTTTCC-3'

C3	F:5'-ACCCGGATAAAGAGGACGGA-3'	R:5'-GTCCGAGCCCGATTTGATGA-3'
JAG1	F:5'-CCCCTGCGAAGTGATTGACA-3'	R:5'-GGGTTGCTCTCACAGTCGTT-3'
CCL5	F:5'-TTTGCCTATATCTCCCGCCC3'	R:5'-TGGTGATAAAGACAACTGCTGC-3'
CXCL2	F:5'-CCGAAGTCATAGCCACTCTC-3'	R:5'-TTCTGTAGGGGCAGGGTCTA-3'
IL6	F:5'-ACTCCATTCGCTGTCTCCCTG-3'	R:5'-TCGCCTGATTGAACCCAGATTG-3'
IL8	F:5'-ATGACTTCCAAGCTGGCTGTTG-3'	R:5'-TTTCATGGATCTTGCTTCTCAGC-3'
TNF-α	F:5'-TCCAGAAGTTGCTTGTGCCT-3'	R:5'-GTTGATGTCGGCTACAACGTG-3'
GAPDH	F:5'-ACGGCACAGTCAAGGCAGA-3'	R:5'-GTGATGGCGTGGACAGTGG-3'
